# Cerebral Salt Wasting Syndrome Following Right Occipital Craniotomy in a Patient With Metastatic Lung Adenocarcinoma

**DOI:** 10.7759/cureus.42271

**Published:** 2023-07-21

**Authors:** Derek Ugwendum, Arnold E Onana, Sai Dheeraj Gutlapalli, Ikpechukwu J Okorie, Abdul Aziz Habib Ullah, Muhammad Khalid Tahir, Farhang Ebrahimi, Jay Nfonoyim

**Affiliations:** 1 Internal Medicine, Richmond University Medical Center, Staten Island, USA; 2 Nephrology, Richmond University Medical Center, Staten Island, USA; 3 Pulmonary and Critical Care, Richmond University Medical Center, Staten Island, USA

**Keywords:** moderate hyponatremia, euvolemic hyponatremia, acute symptomatic hyponatremia, treatment of hyponatremia, cerebral salt-wasting syndrome

## Abstract

Cerebral salt wasting syndrome (CSW) is characterized by excessive natriuresis leading to hyponatremia and hypovolemia. It is commonly encountered among patients who have undergone brain trauma or subarachnoid hemorrhage. The occurrence of CSW after neurosurgical procedures has been frequently reported in the pediatric age group; however, it is a rare phenomenon in adults. We describe the case of a 59-year-old female who developed symptoms of polyuria and polydipsia after a right occipital craniotomy.

## Introduction

Hyponatremia is defined as a serum sodium (Na) concentration of less than 135 mmol/L. It is the most common electrolyte disorder in clinical practice [1]. In patients with neurological disorders, the incidence of hyponatremia is about 50% [2]. It has several etiologies, one of which is a cerebral salt-wasting syndrome (CSW). In the early 1950s, the mechanism of CSW was hypothesized [1]. It is often seen following injury to the central nervous system and has also been reported in patients with subarachnoid hemorrhage [3]. Neuro-meningeal tuberculosis, metastatic carcinoma, and cranial trauma [4-6].

Two hypotheses have been suggested to explain serum Na loss in this condition. The first is a decrease in activation of the sympathetic nervous system that stimulates the juxtaglomerular apparatus, leading to a decrease in renin and aldosterone, causing a subsequent loss of serum Na and water [3]. The second is an increase in brain and atrial natriuretic peptides that increase in certain brain injuries, which leads to serum Na loss by the kidneys [1]. CSW should always be thought of as a cause of hyponatremia, especially in patients who have undergone neurosurgery and should be differentiated from the syndrome of inappropriate antidiuretic hormone (SIADH), as the management of these conditions is entirely different despite the significant overlap in clinical presentation. We present the case of a patient who underwent neurosurgery for an occipital mass seen on computed tomography (CT) and magnetic resonance imaging (MRI) scans of her brain. Following surgery, she developed significant hypotonic hyponatremia, indicating CSW.

## Case presentation

History of presentation

**Figure 1:**
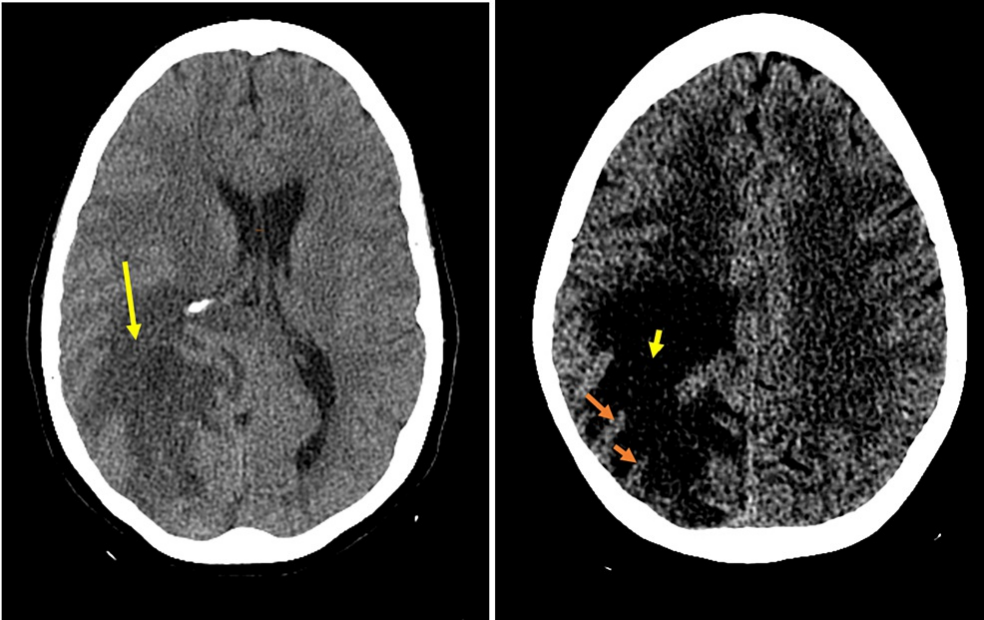
Initial CT scan of the head showed hypodensity within the right parietotemporal and occipital lobes with preservation of the gray-white differentiation [Orange arrows], consistent with vasogenic edema [Yellow arrow]. In addition, there is a mass effect with a midline shift of approximately 7 mm. These findings are concerning for the neoplastic process.

**Figure 2:**
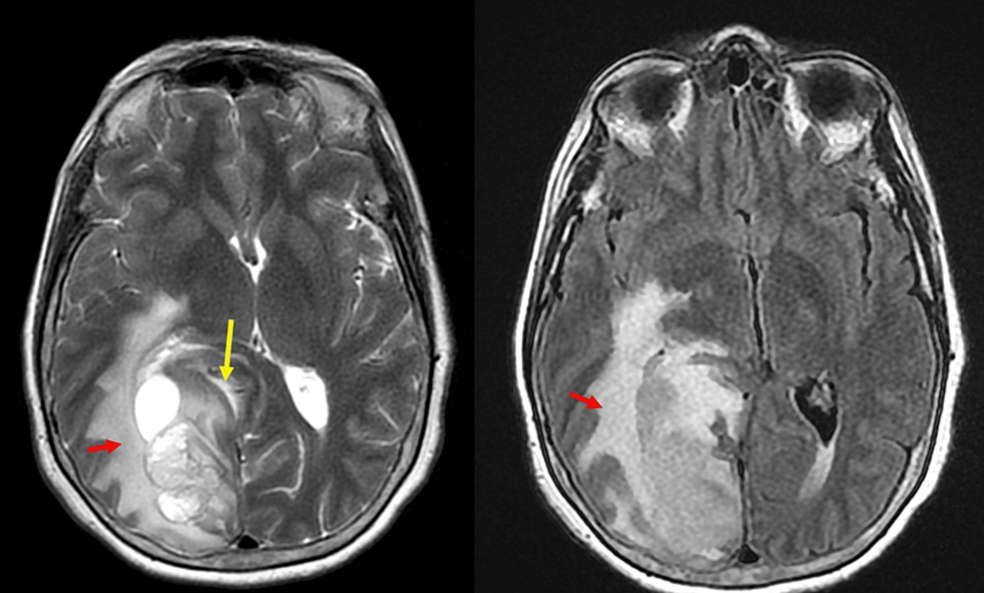
Initial axial T2 propeller (Left) and axial T2 flair (Right) MRI of the head showing a large T2 hyperintensity with poorly defined edema [red arrow], with mass effect in the right posterior parietal and occipital lobes. This corresponds to the area of vasogenic edema on the CT (Figure 1). There is also compression of the posterior horn of the right lateral ventricle [yellow arrow].

**Figure 3:**
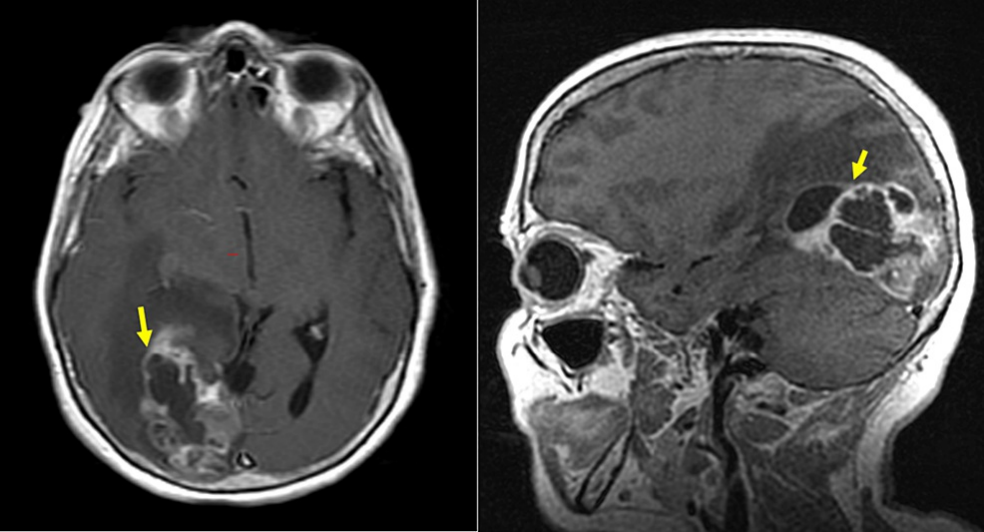
Initial MRI axial T1 SE post-contrast and sagittal T1 post-contrast of the head shows a peripherally enhanced complex mass with areas of necrosis in the parietotemporal and occipital regions, measuring approximately 6.4 x 3.2cm [yellow arrow]. There is a minimal leftward midline shift measuring roughly 8mm.

A 59-year-old female with a past medical history of lung cancer diagnosed in 2017 with metastasis to the left hip post chemotherapy and radiotherapy presented to the emergency department (ED) with a headache for three weeks, not responding to acetaminophen. On admission, her vitals were: Blood pressure (BP) 123/74 mmHg, respiratory rate (RR) 18 breaths/minute, heart rate (HR) 104 beats/minute, breathing on room air saturating at 99%. The patient was not in any acute distress. Her neurological examination was unremarkable. A CT of the head without contrast showed diffuse edema within the white matter of the right temporal, posterior parietal, and occipital lobes and slight compression of the right lateral ventricle with a midline shift of 4.9 mm (Figure 1). A differential diagnosis based on her CT head findings was an intracranial mass suggestive of meningioma, ependymoma, or metastasis in the brain parenchyma. A CT of the thoracic spine showed multiple nodules, which raised suspicion of metastatic lung cancer. An MRI of the head revealed a large peripherally enhancing mass in the posterior parietal and occipital lobes with solid and chronic components, possibly due to metastatic lung cancer. In addition, a large area of vasogenic edema and mass effect in the right posterior and occipital lobes were also seen with compression of the posterior horn of the right lateral ventricle (Figures 2, 3).

The timeline of the pre-and post-surgical period, blood pressure, and laboratory values are shown in Table 1. 

**Table 1:** Investigations Serum Na: Serum sodium, BP: Blood pressure, U Na: Urine sodium, U Osm: Urine osmolarity, S Osm: Serum osmolarity, UO: Urine output

Investigations		Trends								
	12/12	12/13/ Day of Surgery	12/14	12/15	12/16	12/17	12/18	12/19	12/20	12/21
Serum Na (mmol/L)	137	138	136	128	125	133	132	135	136	140
BP (mmHg)	116/70	112/64	122/67	104/74	97/59	99/65	101/60	121/72	101/72	118/78
U Na (mmol/L)	-	-	-	232	>300	208	77	102	106	101
U Osm (mOsm/kg)	-	-	-	877	767	795	699	303	306	420
S Osm (mOsm/kg)	-	-	-	259	265	277	278	279	268	270
UO (mL)	1800	1500	2000	3895	3100	3045	1625	4200	2500	2000

Management

The neurosurgery and oncology teams were consulted. She underwent a right occipital craniotomy indicated for resection of her right occipital mass. Tissue for pathology was collected, which showed metastatic lung adenocarcinoma. Pathology results revealed tumor cells positive for thyroid transcription factor 1 (TTF1) by immunostaining. After surgery, she was placed on Dexamethasone 8 mg daily IV. The patient’s post-surgical course was complicated by polydipsia, hyponatremia with polyuria with a very high urine osmolarity, and hypotension. On a postoperative day two (POD 2), her basic metabolic panel was significant for hyponatremia (drop in serum Na from 136 to 128 mmol/L), serum osmolarity of 259 mOsm/kg, urine osmolarity 877mOsm/kg, a high uric acid level of 8.4 mg/dL, and a urine output of more than 3L/24hr, for which 3% saline was started at a rate of 75 ml/hr. Her serum Na dropped to 125, and she was hypotensive (97/59 mmHg) while on 3% saline. On POD 3, the endocrinology and nephrology teams were consulted for persistently low serum Na levels of 133 mmol/L despite being on 3% normal saline. She was diagnosed with cerebral salt wasting, with a differential diagnosis of SIADH. Her regimen was modified with the addition of a salt tablet dosed at 2,000 mg every 12 hours, 0.9% normal saline with intermittent use of 3% hypertonic solution, and daily monitoring of urine output, blood pressure, serum Na levels, urine, and serum osmolarity (Table 1). Fludrocortisone 0.1 mg every 12 hours was added, and the patient’s serum sodium levels started trending upward to 135 mmol/L on POD 5. Thereafter, her serum Na levels remained within the normal range of 135-145 mmol/L. After stabilizing her medical condition, she was discharged on POD 11 with a radiation oncology referral on an outpatient basis.

## Discussion

Clinically, patients with CSW may present with signs and symptoms such as hyponatremia, muscle cramps, vomiting, headache, orthostatic hypotension, and confusion. Severe symptoms such as shock and coma have also been reported [7].

There are four criteria for the diagnosis of cerebral salt wasting: brain injury, hypovolemia, hypotonic hyponatremia, and natriuresis [8]. Our patient met all four of the aforementioned criteria. In addition, the N-terminal prohormone of brain natriuretic peptide (t) can be used to differentiate SIADH from CSW. It is a good marker in patients with CSW, with a cutoff value of 125 pg/ml. CSW has a positive predictive value of 93.33% [9]. Treatment outcomes for patients with CSW with an etiology other than subarachnoid hemorrhage are usually good. In our patient, we did not measure NTproBNP levels. Mild neurologic deficits may persist in some patients even with optimal treatment [2,10]. Serum uric acid is another marker that can help diagnose CSW. It is elevated as it is a prerenal cause of hyponatremia with hypovolemia [11]. In our patient, the serum uric acid level was recorded at 8.4 mg/dL, confirming that her etiology of hyponatremia was indeed due to CSW.

SIADH is the main differential diagnosis for CSW. It is important to differentiate both, as their management has no overlap. In SIADH, the patients present with hypotonic hyponatremia, an absence of hypovolemia, a normal urine volume, and normal NTproBNP levels. In patients with CSW, they are hypovolemic with increased urine volume and NT pro-BNP levels [1,12].

CSW management depends on its severity and involves the correction of hypovolemia and hyponatremia [13]. If symptoms are mild, 0.9% isotonic saline is administered. With moderate to severe symptoms, 3% hypertonic saline without overcorrecting Na by more than 10 mmol/L within the first 24 hours is recommended [13]. Fludrocortisone has shown effectiveness in treating CSW, especially in cases that are secondary to neuromeningeal tuberculosis and SAH. The dose of fludrocortisone used for management ranges between 0.1 and 0.4 mg [13,14].

Our patient was treated initially with intermittent hypertonic 3% saline solution and 0.9% normal saline at a rate of 75 ml/hr. She was still hyponatremic, and on POD 5, fludrocortisone 0.1 mg every 12 hours was added to her treatment regimen. Her sodium level normalized until she was discharged.

In patients that present with hyponatremia following neurosurgery or brain injury, it is always important for us to differentiate CSW from SIADH. In SIADH, treatment requires fluid restriction, whereas, in CSW, fluid restriction may lead to hypovolemic shock.

## Conclusions

There is considerable overlap in the clinical presentation of CSW and SIADH. Therefore, identifying and differentiating both conditions is important, as their management strategies are different. In patients that present with hyponatremia following neurosurgery or brain injury, as in our case, it is extremely important for us to think of CSW as its management diverges significantly from the treatment of SIADH. In SIADH treatment, fluid restriction is an essential part, whereas in CSW, fluid restriction may lead to hypovolemic shock and a higher risk of mortality. 
